# Formulation, physicochemical characterization and in vitro evaluation of human insulin-loaded microspheres as potential oral carrier

**DOI:** 10.1007/s40204-017-0072-z

**Published:** 2017-09-01

**Authors:** Gauravkumar R. Agrawal, Pravin Wakte, Santosh Shelke

**Affiliations:** 1 Department of Chemical Technology, Dr. Babasaheb Ambedkar Marathwada University, Aurangabad, Maharashtra 431004 India; 2 Department of Pharmaceutics, Yash Institute of Pharmacy, Bajaj Nagar, Aurangabad, Maharashtra 431134 India

**Keywords:** W/O/W multiple emulsion, Eudragit S-100, Microspheres, Controlled release, Oral insulin, Protease inhibitors

## Abstract

**Abstract:**

The objective of the present investigation was to formulate and characterize the human insulin entrapped Eudragit S100 microspheres containing protease inhibitors and to develop an optimized formulation with desirable features. A w/o/w multiple emulsion solvent evaporation technique was employed to produce microspheres of human insulin using Eudragit S-100 as coating material and polyvinyl alcohol as a stabilizer. The resultant microspheres were evaluated for drug-excipient compatibility, encapsulation efficiency, particle size, surface morphology, micromeritic properties, enteric nature, and in vitro drug release studies. Micromeritic properties indicated good flow properties and compressibility. In present investigation formulation F6 with drug/polymer ratio (1:100) was found to be optimal in terms of evaluated parameters where it showed a significantly higher percentage of encapsulation efficiency (76.84%) with minimal drug release (3.25%) in an acidic environment. The optimized formulation (F6) also possessed good spherical shape and particle size (57.42 µm) required to achieve the desired in vitro drug release profile at pH 7.4. The results confirmed that human insulin-loaded Eudragit S-100 microspheres containing protease inhibitor possessed good encapsulation efficiency, pH dependant controlled release carrying encapsulated insulin to its optimum site of absorption. This ultimately resulted in enhanced insulin absorption and biological response.

**Graphical Abstract:**

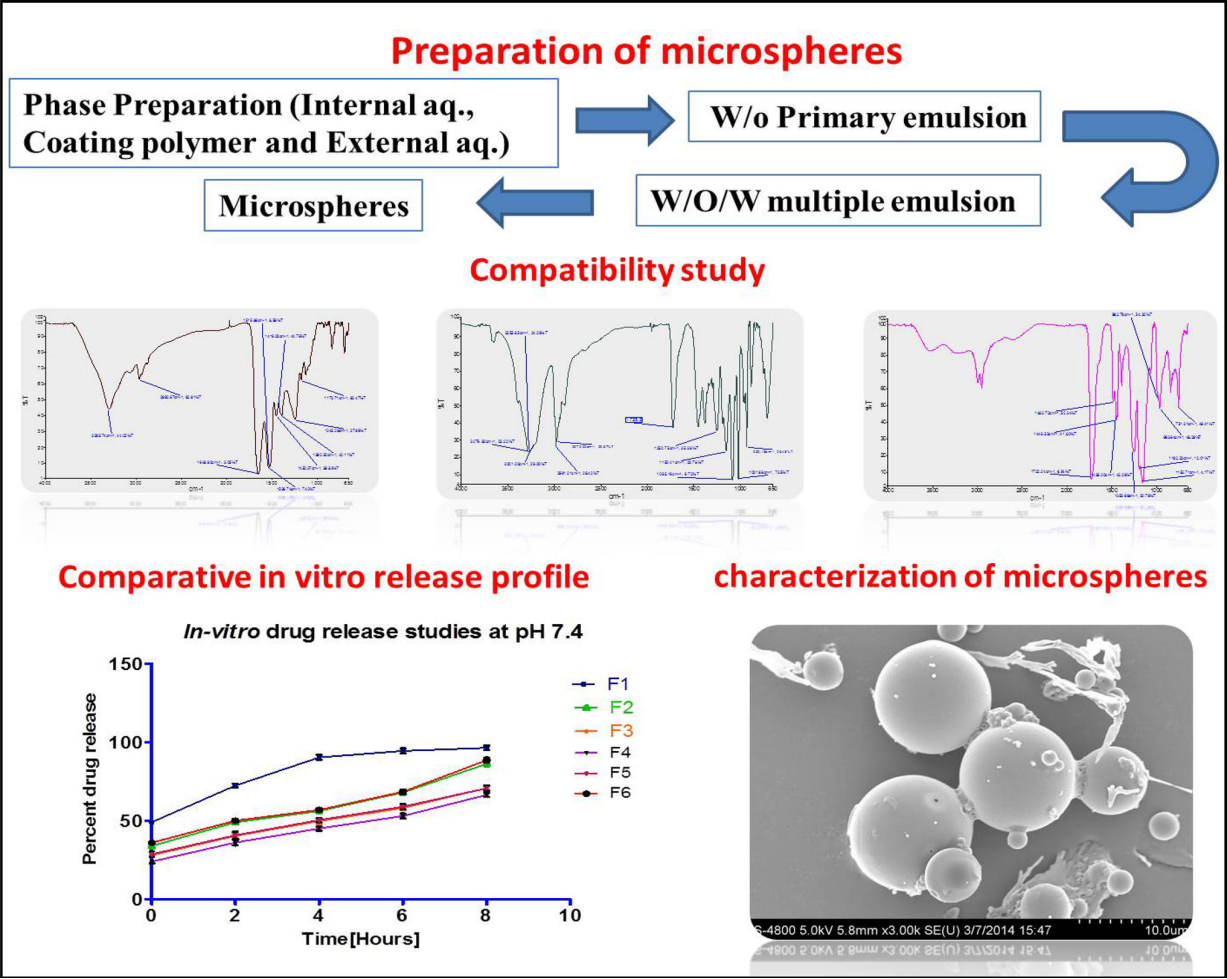

## Introduction

In the past few decades, a significant interest has been focused in formulation and delivery of proteins and peptide drugs for various diseases. A sophisticated pharmaceutical technology is required for production development of these types of drugs, not similar to conventional drugs. Insulin is the most important drug candidate, which is well characterized amongst the therapeutic proteins and peptides derived from recombinant DNA technology. Insulin was isolated from bovine pancreas for the first time in 1922 by Frederick Banting and Charles Best. The aim for delivering exogenous insulin in patients with diabetes is to imitate as closely as possible the normal physiological insulin secretion as in healthy humans (George and Abraham 2006). Conventional insulin treatment is basically a replacement therapy for the management of diabetes, wherein insulin is exogenously administered by the subcutaneous route to secrete it as in non-diabetic pancreatic secretion. Insulin delivery by subcutaneous route has been extensively used and explored until now. Parenteral routes are satisfactory in terms of efficacy in the great majority of cases. However, they can result in peripheral hyperinsulinemia, the stimulation of smooth muscle cells proliferation, and the incorporation of glucose into the lipid of arterial walls, which can cause diabetic micro and macroangiopathy (Adameova and Dhalla 2014). In addition to this, pain of daily injections, physiological stress, inconvenience, cost, risks, infection, inability to handle insulin, and the localized deposition of insulin, leading to local hypertrophy and fat deposition at the injection sites are some of the problems for administration by parenteral route (Khafagy et al. 2007). With the advancement of recombinant DNA technology, it has become conceivable to develop well-defined recombinant human therapeutic proteins (Torosantucci et al. 2014). Due to its synthesis, insulin obtained by recombinant DNA technology, large quantities of insulin crystalline powders are available at an affordable price. This is the most important factor that has made insulin to be one of the most popular proteins to be studied for non-parenteral deliveries. Consequently, the results of research into several aspects of the delivery of the insulin are available. These days, there has been a lot of interest in the investigation and feasibility of non-invasive routes for insulin delivery and their product developments in the pharmaceutical industry. These non-invasive routes (Khafagy et al. 2007) include oral (Al-Remawi et al. 2017), nasal (Bloch et al. 2017), buccal (Kumria and Goomber 2011), pulmonary (Pfützner and forst 2005), transdermal (Leeladurga et al. 2016), ocular (Lee et al. 2002) and rectal (du Plessis et al. 2010) drug delivery systems (Owens 2002; Cefalu 2004).

Despite the different approaches being studied the oral delivery is considered the significant choice by researchers owing to the ease of oral administration and greater patient compliance. However, there are several reasons that cause reduced oral bioavailability of peptides and proteins. Major drawback identified with the oral delivery is the harsh environment of the gastrointestinal (GI) tract due to the presence of proteolytic enzymes, acidic and basic pH environment which is responsible to degrade proteins and peptides into their constituent amino acids. In addition to enzymatic barriers, physical barriers also contribute difficulty in bioavailability of orally administered protein drug (Goldberg and Gomez-Orellana 2003). Novel drug delivery strategies have increased the bioavailability of orally administered insulin (Muheem et al. 2016).

There is a need of delivering protein drug in a convenient and effective manner to implement targeted drug delivery systems. Despite various challenges, a significant progress has been made in convenient, non-invasive delivery of proteins and peptides through specific routes of administration. The targeted delivery of proteins and peptides to specific sites of action has the advantage of lowering the total dose to be delivered and to concentrate the therapeutic dose at specific sites of pharmacological action (Pettit and Gombotz 1998; Shelke et al. 2016). Absorption is not uniform throughout the GIT due to differences in composition and thickness of the mucus layer, pH, surface area, and enzyme activity (Ashford 2017). Therefore, colon-targeted drug delivery for therapeutic proteins, has several merits like prolonged residence time, reduced enzymatic activity, increased tissue responsiveness to absorption enhancers, and natural absorptive characteristics (Morishita and Park 2009). Oral administration of drugs provides the dual advantage of site specific local delivery to the superficial layers of the GIT and systemic delivery to the blood and lymphatic systems. Oral administration of insulin mimics the natural insulin release from the pancreas (first to the portal vein, then to the liver, followed by the peripheral circulation). Research has revealed the abnormality such as peripheral hyperinsulinemia due to administration of insulin through other routes, which in turn may lead to micro- and macro-vascular diseases (Babu et al. 2008). However, the harsh hydrolytic environment of the GIT and the epithelial barriers to absorption are still major challenges to the success of this mode of administration of peptide and protein drugs.

Microencapsulation of proteins into microspheres has several concerns in comparison to peptides (Yuan et al. 2009). Because proteins have structural complexities as characterized by a well-defined tertiary structure which is usually affected by the harsh manufacturing conditions of the conventional emulsification process used for microsphere preparation (Freiberg and Zhu 2004).

Colon-targeted delivery systems for the administration of insulin have been studied extensively over the past few years (Maroni et al. 2016). Insulin is a polypeptide hormone (MW 5808 Da). It consists of two chains of amino acids, termed as chain A (21 amino acids) and chain B (30 amino acids). These chains are interconnected by two inter-chain disulfide bridges to stabilize its globular three-dimensional conformations with prevailing polar exterior and hidden hydrophobic residues (Babu et al. 2008). In addition, a further intra-chain disulfide bridge is present within a chain. Insulin shows a marked tendency towards self-association to form soluble dimers and hexamers (Hostrup et al. 2009). The latter consists of three dimers arranged around two divalent zinc cations. Insulin as a hormone is stored in a hexameric form in the body where, they are converted to monomers when the concentration reduces in contact with biological fluids or so.

In the present investigation, attempts were made to microencapsulate human insulin in Eudragit S100 microspheres containing protease inhibitors and to study their drug loading and in vitro release profile. Eudragit S-100, a commonly employed polymer was selected for the controlled oral delivery whereas, human insulin a model therapeutic protein was used for encapsulation by w/o/w multiple emulsion solvent evaporation method.

## Materials and methods

### Materials

Human insulin, methacrylic acid copolymer (Eudragit S100) and Aprotinin (recombinant bovine) were obtained from the Wockhardt Research Centre, Aurangabad (M.S.), India. Hydrochloric acid and sodium sulfate were procured from Merck, India and whereas, acetonitrile from J T Baker, India. All the chemicals and reagents were of analytical grades and solvents were HPLC grade.

### Methods

#### Preparation of microspheres

Human insulin-loaded Eudragit S100 microspheres containing protease inhibitor was prepared by w/o/w multiple emulsion solvent evaporation technique (Das et al. 2015).

#### Preparation of phases (inner aqueous, coating polymer and outer aqueous)

Qualitative and quantitative compositions (Table 1) of insulin-loaded microspheres were used for designing the formulation. Human insulin was dissolved in a small quantity of purified water by adding 1 N hydrochloric acid (drop-wise) until the formation of clear solution. A required quantity of zinc oxide solution from zinc stock solution (1% w/v) was added to this concentrated insulin solution to obtain a final concentration of 10 µg zinc ions/mg insulin. Aprotinin from its stock solution (100 µg/ml) was also added into the insulin–zinc solution to acquire the final concentration of 50 ng aprotinin/mg insulin. Furthermore glycerol was added to the above prepared solution (IAP). Eudragit S-100 was dissolved in organic solvent mixtures (5:6:4) of dichloromethane, ethanol and isopropyl alcohol (coating polymer organic phase). A required quantity of polyvinyl alcohol was added into hot purified water under constant stirring and allowed to dissolve completely. Volume was made up with purified water (outer aqueous phase).

**Table 1:** Composition of controlled oral delivery system of human insulin

Phase I: internal aqueous phase				
Sr. no.	Ingredients	Quantity/ml	Batch quantity/400 µl	Function
1.	Human insulin (r-DNA origin)	120 mg	48 mg	Active ingredient
2.	Glycerol	50 mg	20 mg	Stabilizer
3.	Zinc as zinc oxide	1.2 mg	0.48 mg	Stabilizer
4.	Aprotinin	6.0 µg	2.4 µg	Protease inhibitor
5.	Hydrochloric acid	Q.S. to pH	Q.S. to pH	pH adjustment
6.	Purified water	Q.S. to 1.0 ml	Q.S. to 400 µl	Vehicle

Phase II: coating material organic phase				
Sr. no.	Ingredients	Quantity/ml	Batch quantity/80 ml	Function
7.	Methacrylic acid copolymer (Eudragit S-100)	60 mg	4.8 g	Coating material
8.	Dichloromethane	0.3332 ml	26.66 ml	Solvent
9.	Ethanol	0.4 ml	32 ml	Solvent
10.	Isopropyl alcohol	0.2665 ml	21.32 ml	Solvent

Phase III: external aqueous phase				
Sr. no.	Ingredients	Quantity/ml	Batch quantity/200 ml	Function
11.	Polyvinyl alcohol	10 mg	2.0 g	Stabilizer
12.	Purified water	Q.S. to 1.0 ml	200 ml	Vehicle

#### Preparation of primary and multiple emulsions

Primary emulsification was performed by a homogenizing inner aqueous solution with the coating polymer solution. Both phases were placed in the beaker in an ice bath for 1 min and homogenized at 10,000 rpm using high speed homogenizer to form the primary emulsion (w/o). Furthermore, secondary emulsification was performed in a plastic beaker using high speed homogenizer. Primary emulsion was added slowly drop-wise to the outer aqueous phase (EAP) under constant stirring (5000 rpm) at ambient temperature for 5 min to obtain a multiple emulsion (w/o/w).

#### Formation of microspheres

Microspheres were formed by solvent evaporation technique. Resulting emulsion mixture was stirred at 250 rpm at an ambient temperature for 18–24 h to allow solvent evaporation leading to formation of microspheres. Centrifugation (9000 rpm) was performed for 20 min to collect hardened microspheres which were further washed three times with purified water for 5 min each. The obtained supernatant was filtered through a coarse filter to obtain microspheres retained in solution. Finally, the resulting microspheres were freeze-dried by adding 1% w/v of mannitol for 24 h, collected and stored.

*Note* Exactly same experimental procedure of microspheres fabrication was adopted for all trial run batches with the exceptions of quantities of recombinant human insulin, Eudragit S100 and polyvinyl alcohol as per Table 2. The batch composition (Table 1) and procedure were designed by taking into consideration F6 trial run, which is an optimal formulation based on data available.

**Table 2:** Formulation details of trial run batches during development

Formulation codes	Drug:polymer ratio	Internal aqueous phase (W_1_)	Coating polymer oil phase (O)	External aqueous phase (W_2_)
		Human insulin (mg)	Eudragit S100 (g)	Polyvinyl alcohol (g)
F1	1:60	24	1.44	1.0
F2	1:100	24	2.40	1.5
F3	1:150	36	5.40	2.0
F4	1:200	48	9.60	2.0
F5	1:150	48	7.20	2.0
F6	1:100	48	4.80	2.0

### Characterization of emulsified human insulin-loaded microspheres

#### Drug-excipients compatibility study by FTIR spectroscopy

Compatibility studies were confirmed by Fourier transform infrared spectroscopy (FTIR) using the KBr pellet technique (Shelke et al. 2015). The range of 650–4000 cm^−1^ was assessed to identify any possible interaction between the drug and polymer. The samples of the pure drug, polymer and optimized microsphere formulation were subjected to analysis separately. Pure drug, polymer and formulation were dispersed in KBr powder and the pellets were made by applying 6000 kg/cm^2^ pressure. A physical mixture was prepared and the samples were analyzed by Shimadzu IR Spectra Analyzer (Shimadzu, Japan). FTIR spectra were obtained by powder diffuse reflectance on Fourier transform infrared spectrophotometer at room temperature using Perkin Elmer Spectrum 88522.

#### Production yield

The production yield (%) was calculated after weighing the comprehensively freeze-dried microspheres, known as practical yield using the following formula:

$$ {\text{Production yield}}\,(\%) = \frac{{{\text{Practical yield}}}}{{{\text{Theoretical yield}}}}*100 $$

#### Particle size

Particle size was determined by optical microscopic method (Trivedi et al. 2014) with minor modifications using phase contrast microscope equipped with camera (Olympus microscope with Nikon camera). A small amount of freeze-dried microspheres was suspended in 5 ml of purified water and subjected to ultrasonication for 5 s. One drop of resulting suspension was placed on clean glass slide and mounted on stage of microscope. A total of 100 microspheres were measured for particle size using a calibrated ocular micrometer at 100× objective and mean diameter was reported.

#### Micromeritic properties

The micromeritic evaluation of microspheres was done to estimate the flow properties, packing properties and porosity of powder, obtained to determine their suitability for table formation or capsule filling. A weighed quantity of microspheres was transferred into a graduated cylinder from each batch to determine the bulk and tapped density using USP-I tapped density tester (TD 1025. Lab India Instruments, Mumbai, India). A fixed funnel method was used to study the angle of repose (*θ*). Bulk characterization parameters were selected to study the flow properties by the following formulas.

- $$ {\text{Bulk density}} \left({\frac{\text{g}}{\text{mL}}} \right) = \frac{{{\text{Weight of microspheres}}}}{\text{Volume}} $$
- $$ {\text{Tapped density}} \left( {\frac{\text{g}}{\text{mL}}} \right) = \frac{{{\text{Weight of microspheres}}}}{\text{Volume}} $$
- $$ {\text{Carr's index}} = \frac{{{\text{Tapped density}} - {\text{bulk density}}}}{\text{Tapped}}*100 $$
- $$ {\text{Hausner's}}\;{\text{ratio}} = \frac{{{\text{Tapped}}\;{\text{density}}}}{{{\text{Bulk}}\;{\text{density}}}} $$
- $$ {\text{Tan}}\,\theta = h/r $$

#### Encapsulation efficiency

Microspheres were added to ethanol and stirred until complete dissolution. Phosphate buffer saline (pH 7.4) was added to ethanol solution and mixed thoroughly. Mixture was allowed to stand for 30 min at room temperature. The prepared mixture was then acidified with 9.6 N hydrochloric acid and centrifuged (3000 rpm) for 10 min, at room temperature. The resulting supernatant was analyzed for insulin content by “reverse phase-high performance liquid chromatography” (RP-HPLC). Encapsulation efficiency (%) was calculated using the following formula,

$$ {\text{Encapsulation efficiency}}\,(\%) = \frac{{{\text{Actual insulin loading}}}}{{{\text{Theoretical insulin loading}}}}*100 $$

#### Enteric nature of microspheres

An investigation was performed to confirm the retention of drug in the acidic environment of stomach at pH 1.2. Formulated microspheres were dispersed in 0.1 N HCl for 2 h, which was already equilibrated at 37 °C ± 0.5. The sample was subjected to centrifugation at 3000 rpm for 10 min, at room temperature and the insulin content in the supernatant (after 0.22 µ filtration) was analyzed by U-HPLC.

#### In vitro drug release at pH 7.4

To understand the effect of protease inhibitor, the in vitro release of insulin from microspheres was evaluated in phosphate buffer (pH 7.4) containing bovine trypsin at the weight ratio of 200:1 (human insulin to enzyme). In a control experiment, insulin solutions without encapsulation were also subjected to study enzyme degradation. The prepared microspheres were incubated in an enzyme free dissolution media under the same conditions.

In vitro release testing study was conducted in six replicates using in-house dissolution apparatus. It was provided with plastic tubes of 15 ml capacity mounted on gel rocker, providing necessary agitation for sufficient mixing of dissolution media without any frothing of insulin. Microspheres of 100 mg were transferred to each plastic tube containing pre-warmed dissolution media (10 ml) and maintained at 37 ± 0.5 °C under agitation with 25 oscillations/min on gel rocker at 25–30° angle of inclination. An aliquot of 500 µl was withdrawn every 2 h up to 8 h at the interval of 0, 2, 4, 6 and 8 h. The volume was replaced immediately by fresh phosphate buffer at each sampling time interval. Complete homogeneity of suspension was ensured prior to sampling at different time intervals. The samples withdrawn were centrifuged at 3000 rpm for 10 min at room temperature. The supernatant obtained after 0.22 μ filtration was acidified with 1.0 µl of 9.6 N HCl for ~250 µl of supernatant and the percent insulin release was estimated by U-HPLC method.

#### Surface morphology of microspheres

The surface morphology of insulin microspheres was examined using Field emission scanning electron microscopy FE-SEM (Hitachi S-4800, Japan). The prepared microspheres were evaluated for shape, size and surface characteristics. Prior to observation, freeze-dried microspheres were placed on an adhesive stub. These microspheres were further coated with gold–palladium under vacuum using an ion-coater. The coated samples were then examined under the microscope at 5 kV and photographed.

## Results and discussion

### Preparation of microspheres

Human insulin-loaded Eudragit S100 microspheres were prepared by w/o/w multiple emulsion solvent evaporation technique. Microsphere formation was initiated by preparing the inner aqueous, coating polymer and outer aqueous phases. Phase preparation was followed by formation of primary and multiple emulsions. Finally the microspheres were formed by solvent evaporation technique. During the formulation of microspheres organic solvents like dichloromethane and ethanol were allowed to evaporate for about overnight during the process of microencapsulation. Even though there could be trace amounts of these solvents present in microspheres. Considering the stability issues sufficient efforts were made to maintain structural conformation of human insulin in microspheres by selecting right excipients and their levels in inner aqueous phase of microspheres. Zinc stabilizes hexameric state of human insulin, which is supposed to be the most desirable state expected during the development of human insulin-based formulations (Asani et al. 2016). In addition, glycerol was also added to enhance physical stability of human insulin by reducing aggregations during the course of harsh processing conditions of microencapsulation (Soni 2017). This novel formulated composition in presence of glycerol and zinc oxide in IAP has helped in reducing protein denaturation during microsphere fabrication.

### Drug-excipients compatibility study by FTIR spectroscopy

**Fig. 1:**
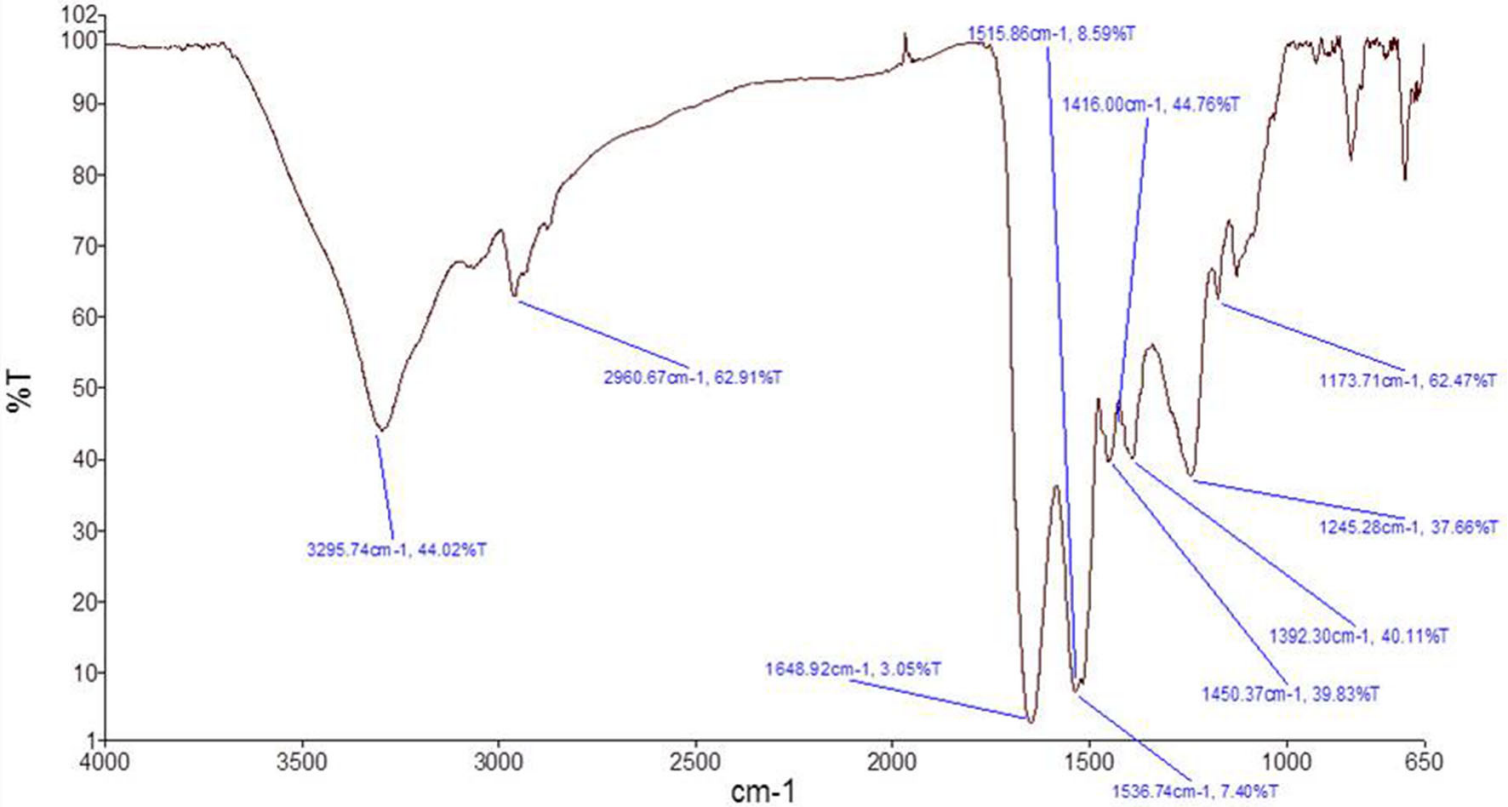
FTIR spectra of pure human insulin

**Fig. 2:**
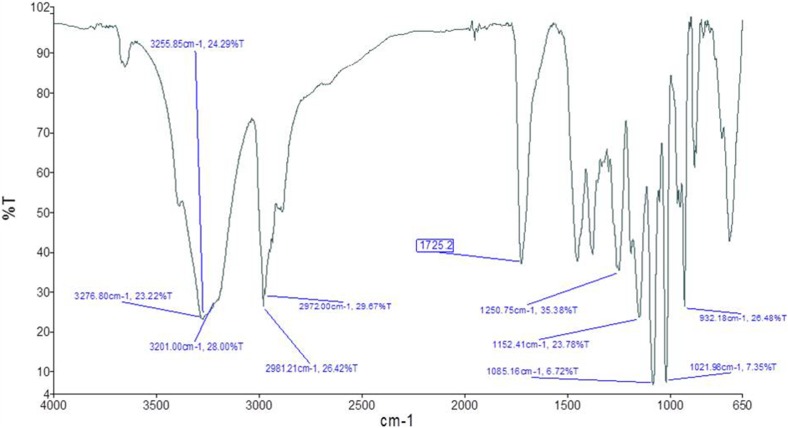
FTIR spectra of eudragit S100

**Fig. 3:**
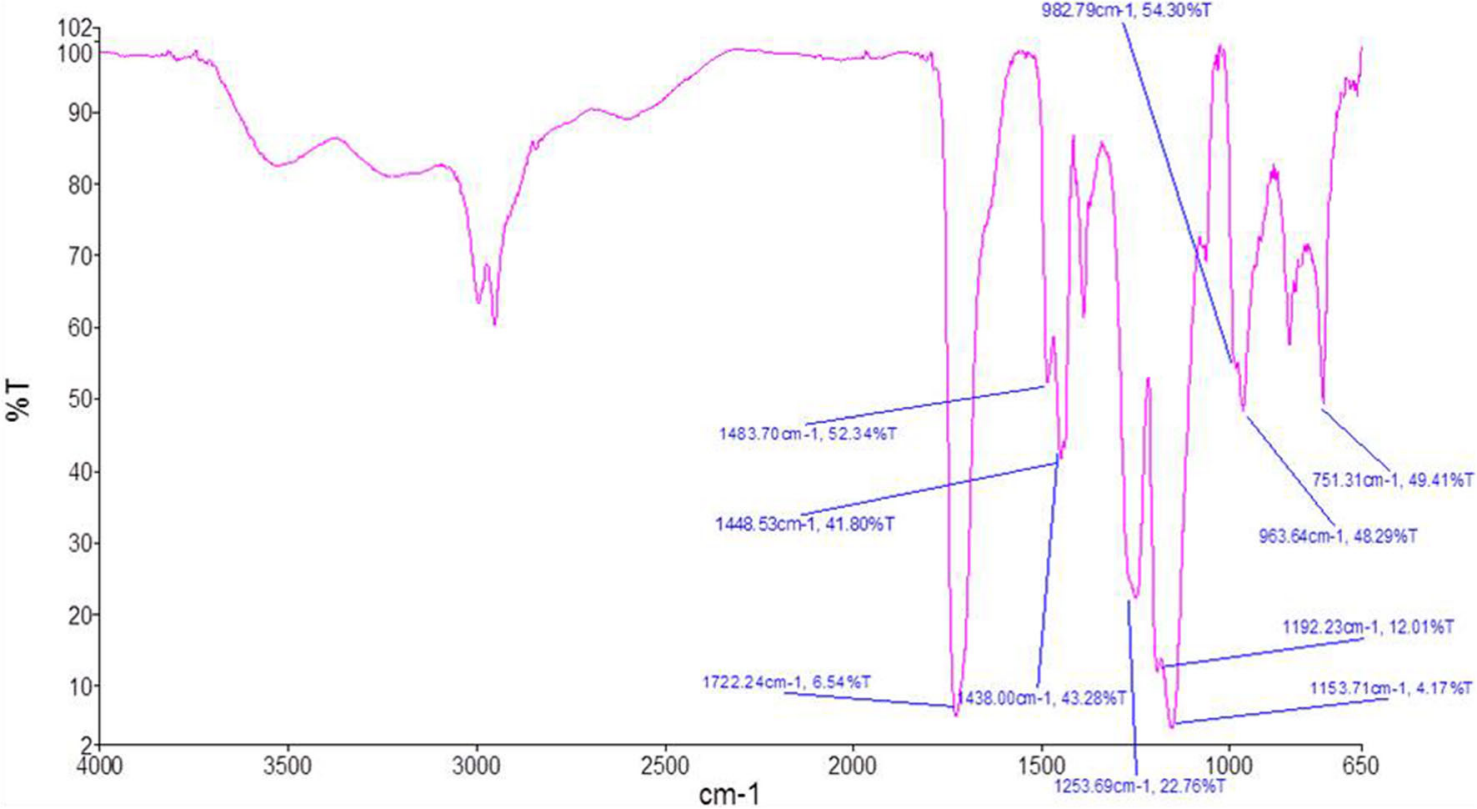
FTIR spectra of human insulin loaded microsphere formulation

Drug-excipients interaction studies by FTIR spectroscopy revealed no physical interaction, suggesting the compatibility of drug and the excipients used in the formulation. FTIR spectra for recombinant human insulin and Eudragit are illustrated in Figs. 1 and 2, respectively. As recorded in Fig. 1 characteristic peaks of drug indicates no major shift of the peak positions, matching the formulation spectrum (Fig. 3). Thus, the record indicates compatibility of the drug and excipients based on comparability of frequencies for critical functional groups as shown in Table 3.

**Table 3:** FTIR data interpretation

Functional group	Frequency for drug (cm^−1^)	Frequency for formulation (cm^−1^)
Amide II (N–H)	1536.7	1448.53
C=O stretching	1648.9	1722.24

The characteristic bands of the C=O stretching vibrations of the esterified carboxyl groups at 1725.2 cm^−1^ is shown in Fig. 2 for Eudragit S100

### Production yield

All the prepared formulations were evaluated for percentage yield (Table 4) and were found to be in the range of 35.16–57.92%. Formulation F6 showed the maximum production yield, whereas F4 formulation reported lowest yield. The low production yield could be due to the handling loss during various steps of processing. These include sticking of the polymeric solution to the glass container, loss of microspheres during the washing step, etc. Sticking loss could be minimized by some plastic or polyethylene based containers.

**Table 4:** Evaluation of microsphere properties

Formulation codes	Production yield (%)	Encapsulation efficiency (%±SD)^a^	Particle size (µm ± SD)^b^	Enteric nature (%±SD)^a^
F1	52.14	36.21 ± 1.45	46.12 ± 16.45	18.57 ± 1.78
F2	47.93	73.42 ± 1.05	28.14 ± 18.35	4.02 ± 1.04
F3	39.66	84.66 ± 1.64	54.44 ± 11.05	2.23 ± 1.35
F4	35.16	92.08 ± 1.24	51.87 ± 13.48	0.85 ± 0.85
F5	41.03	82.54 ± 1.73	42.25 ± 11.52	2.54 ± 1.47
F6	57.92	76.84 ± 1.28	57.42 ± 13.20	3.25 ± 1.12

^a^Mean ± SD (*n* = 3)

^b^Mean ± SD (*n* = 100)

### Particle size

Particle size was determined by optical microscopy using optical trianocular microscope coupled with a camera at 100× objective. The particle size was measured in the range of 10–60 µm among different formulations. A higher concentration of polymer has resulted in more viscous dispersion, which ultimately forms larger droplets and consequently larger microspheres, resulting in increased particle size (Table 4).

### Micromeritic properties

The data obtained for micromeritic properties (Table 5) denotes powder characteristics. Carr’s index was evaluated to determine compressibility characteristics of the microspheres and was found to be in the range of 15.79–19.64% indicating good compressibility. Hausner’s ratio was recorded to be in the range of 1.19–1.24, confirming good flowability of microspheres. Hausner’s ratio greater than 1.25 is generally considered to be an indicator of poor flowability. Angle of repose for various formulations was found in the range of 13.25 ± 0.17–15.68 ± 0.21 showing the free flowing nature of the microspheres. Data recorded for bulk characterization confirm that the formulation F6 can be considered as the optimized formulation, showing good flow property, compressibility putting forward the potential method for the use in large industrial production.

**Table 5:** Micromeritic properties of microspheres

Formulation code	Bulk density (g/ml)^a^	Tapped density (g/ml)^a^	Carr’s index (%)	Hausner’s ratio	Angle of repose (º)^a^
F1	0.45 ± 0.30	0.56 ± 0.08	19.64	1.24	13.25 ± 0.17
F2	0.48 ± 0.18	0.57 ± 0.11	15.79	1.19	14.85 ± 0.19
F3	0.52 ± 0.05	0.63 ± 0.24	17.46	1.21	15.68 ± 0.21
F4	0.51 ± 0.21	0.61 ± 0.04	16.39	1.20	13.54 ± 0.28
F5	0.49 ± 0.12	0.60 ± 0.09	18.33	1.22	15.36 ± 0.12
F6	0.53 ± 0.14	0.64 ± 0.07	17.19	1.21	14.45 ± 0.25

^a^Mean ± SD (*n* = 3)

### Encapsulation efficiency

In one study it has been reported that the optimized ratio of inner aqueous phase (IAP) to oil phase to EAP was 1:100:500, that is, 50 µl of IAP and 5 ml of oil phase and 25 ml of EAP (Jain et al. 2005). It was observed that for higher encapsulation of insulin, a smaller volume of IAP is desirable. Ibrahim et al. (2005) have reported a similar finding while encapsulating insulin in polylactic acid microspheres by double-emulsion solvent evaporation method. In the present investigation formulation F4 showed maximum encapsulation efficiency (92.36% ± 1.24). However, further experiments were carried out to reduce the amount of polymer without significant impacts on encapsulation efficiency and enteric nature of microspheres. In the findings formulation F6 produced good encapsulation efficiency (78.56% ± 1.28) by reducing the amount of polymer almost by 50% with minimal drug release in acidic environment (3.25%) ultimately retaining good spherical shape.

### Enteric nature of microspheres

The enteric nature of the coating was tested on PVA-stabilized microspheres. The absolute enteric coating could not be achieved as formulation F6 showed the drug release of 3.25% in 0.1 N HCl medium (pH 1.2) in 2 h. The reason for this initial burst release might be due to the release of adsorbed insulin molecules from the surface of the microspheres. Another reason could be leaching of insulin molecules from the microspheres, where inefficient coating could have occurred as observed in case of formulation F1. Less than 1% drug was released in 0.1 N HCl in case of F4 formulation where drug to polymer ratio was too high, i.e., 1:200. It has reported that insulin-loaded polymethacrylic acid and polymethacrylic acid-alginate microparticles release around 30% of loaded insulin within 2 h at pH 1.2 (Sajeesh and Sharma 2004). Polymethacrylic acid-alginate microparticles showed burst release of 90% loaded insulin in 1 h at pH 7.4, whereas polymethacrylic acid microparticles exhibited sustained release of insulin for >5 h, at the same pH.

### In vitro drug release at pH 7.4

GI conditions are simulated for gastric and colon conditions by the selection of (i) 0.1 N HCl to evaluate the integrity of microspheres at acidic pH 1.2 and (ii) phosphate buffer saline at pH 7.4 (10 mM) containing bovine trypsin at the weight ratio of 200:1 (human insulin to enzyme) to mimic the presence of gastrointestinal enzymatic degradation.

**Fig. 4:**
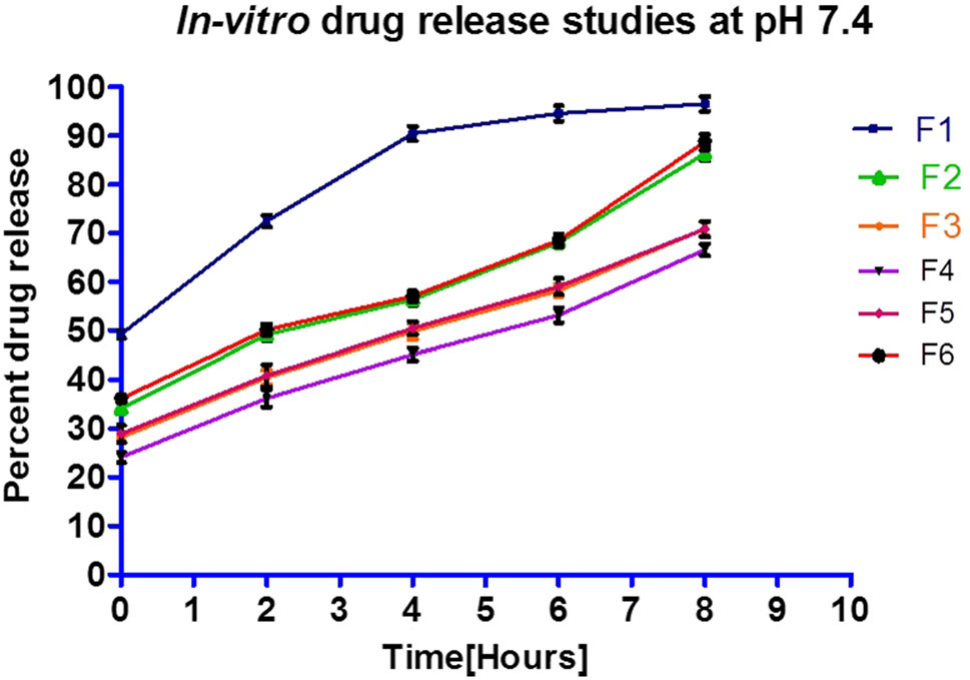
Comparative in-vitro release profile of formulations

The data (not disclosed) obtained for the in vitro release at pH 7.4 depicts the degradation of insulin in presence of trypsin without protease inhibitors. Microsphere formulations indicated increased release of human insulin at pH 7.4 without the presence of trypsin in dissolution medium (Fig. 4). This may be ascribed to slightly less efficiency of aprotinin against trypsin inhibition or slightly higher trypsin concentration selected for the purpose of demonstration.

In the study for microspheres release an initial burst release was recorded in the release profile indicating smaller inner aqueous phase (IAP) responsible for controlling the initial burst release. It was noticed that as the ratio of IAP to coating polymer organic phase (O) was increased from 1:5 to 1:100, the burst release decreased (data not enclosed). This might be due to the possible formation of primary emulsion of smaller volume of IAP resulting in smaller drug globules with less protein on surface which consequently results in minute amount of protein release during the initial hours. On the contrary, in case of microspheres made with larger IAP, large droplets are formed during primary emulsification, which further result in higher burst release due to immediate release of surface associated protein. It has been reported (De Rosa et al. 2000) that microsphere formulations with high quantity of insulin located on or near the surface of microspheres showed higher initial burst release.

The transit time of drug candidate through the absorptive area of the gastrointestinal (GI) tract is between 9 and 12 h (Becker et al. 2014) whereas, gamma scintigraphy studies confirm a short GI transit time from mouth to cecum of 4–6 h (Shargel et al. 2004). Thus, assuming a maximum GI tract transit time of 12 h, a formulation in the cecum is expected to release its drug load within 6 h. Considering this approach, an in vitro drug release from all the batches of microspheres was studied for duration of 8 h (Fig. 4).

Based on in vitro release data (Table 6) it can be concluded that formulation (F4) showed slowest release as it consisted of drug:polymer ratio 1:200 whereas, formulation F1 was found to demonstrate faster release than rest of the formulations as the amount of polymer was not sufficient to retard the drug release. Formulation (F6) consisted of drug:polymer ratio 1:100 with desired slower release profile at pH 7.4 where, encapsulation efficiency was also observed better than other formulations. Loading efficiency (formulation F6) of human insulin was higher in comparison to F2 formulation in which drug:polymer ratio was similar, despite the difference of PVA concentration in EAP. The data obtained suggest that the optimized microsphere formulation (F6) is stable at acidic pH 1.2 and thus proves its enteric nature due to effective Eudragit S 100 coating, which simultaneously would be able to release most human insulin encapsulated at pH 7.4 slowly over the period of eight hand hence proving potential for colon-targeted delivery. As a result, F6 formulation was found to be optimal in terms of desired in vitro release performance and higher drug loading with good encapsulation capabilities.

**Table 6:** In vitro drug release data at pH 7.4

Sampling time points (h)	Cumulative drug release (% ± SD)^a^					
	F1	F2	F3	F4	F5	F6
0	49.40 ± 0.86	34.43 ± 0.42	29.49 ± 0.37	24.34 ± 0.15	29.50 ± 0.17	36.56 ± 0.64
2	72.18 ± 0.97	49.43 ± 0.32	41.53 ± 0.34	36.43 ± 0.18	41.17 ± 0.57	50.41 ± 0.88
4	90.40 ± 1.27	56.84 ± 1.00	50.93 ± 0.31	45.77 ± 0.30	50.65 ± 0.29	57.31 ± 1.11
6	94.55 ± 0.86	68.53 ± 1.12	58.80 ± 0.60	53.97 ± 0.15	59.94 ± 0.90	68.97 ± 0.34
8	96.57 ± 1.52	86.73 ± 0.83	71.88 ± 0.24	66.08 ± 1.04	71.81 ± 0.24	88.78 ± 1.41

^a^Mean ± SD (*n* = 6)

### Surface morphology of microspheres

**Fig. 5:**
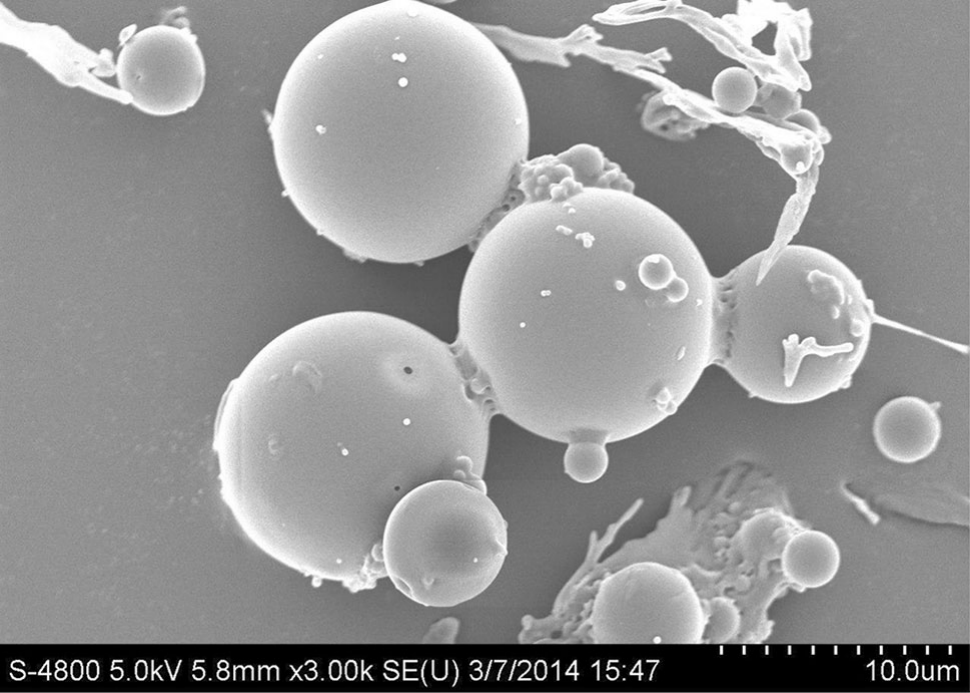
SEM photomicrograph of insulin loaded microspheres

Mode of action for stabilization of microspheres by polyvinyl alcohol in EAP involves association of PVP with the surface of the protein-containing droplets produced during sonication of primary emulsion. As a result of steric and charge effects, coalescence of drug globules is prevented ultimately. Hardening of microspheres occurs due to the polymer precipitation and gradual diffusion of solvent from the polymer solution droplets into the EAP (W2) (Jain et al. 2005). The PVA-stabilized microspheres were subjected to scanning electron microscopy for morphological evaluation. The shapes of microspheres were observed to be spherical, as visible from the photographs. Figure 5 confirms spherical shape for PVA-stabilized (F6) microspheres with smooth surface. Final optimal formulation F6 has a significantly higher percentage of encapsulation efficiency 76.84% with minimal drug release in acidic environment 3.25% and possesses good spherical shape and particle size was found to be 57.42 µm for obtaining a desired in vitro release profile at pH 7.4.

## Conclusion

The data obtained in the present work illustrate satisfactory performance of PVA-stabilized microspheres (F6) with respect to better encapsulation efficiency and delayed in vitro release at neutral pH for recombinant human insulin from Eudragit S100 microspheres. Optimal formulation demonstrates as a potential candidate carrier of insulin in GIT by preventing gastric degradation at pH 1.2 and would release human insulin slowly at pH 7.4 in the colonic region. A way forward to optimum formulation could be for long-term stability under adverse storage conditions of thermo-mechanical stress and the therapeutic effects of this insulin microspheres in vivo. In conclusion, insulin-loaded Eudragit S-100 microspheres containing Aprotinin as protease inhibitor possesses remarkably higher loading efficiency, pH dependent in in vitro release profile.
